# The evolution of disease: anthropological perspectives on epidemiologic transitions

**DOI:** 10.3402/gha.v7.23303

**Published:** 2014-05-15

**Authors:** Molly Kathleen Zuckerman, Kristin Nicole Harper, Ronald Barrett, George John Armelagos

**Affiliations:** 1 Department of Anthropology and Middle Eastern Cultures, Cobb Institute of Archaeology, Mississippi State University, Mississippi State, MS, USA; 2 Department of Environmental Health Sciences, Columbia University Medical Center, New York, NY, USA; 3 Department of Anthropology, Macalester College, Saint Paul, MN, USA; 4 Department of Anthropology, Emory University, Atlanta, GA, USA

**Keywords:** epidemiologic transitions, Omran, epidemiology, hygiene hypothesis, infectious disease

## Abstract

**Background:**

The model of epidemiologic transitions has served as a guiding framework for understanding relationships between patterns of human health and disease and economic development for the past several decades. However, epidemiologic transition theory is infrequently employed in epidemiology.

**Objective:**

Moving beyond Omran's original formulation, we discuss critiques and modifications of the theory of epidemiologic transitions and highlight some of the ways in which incorporating epidemiologic transition theory can benefit theory and practice in epidemiology.

**Design:**

We focus on two broad contemporary trends in human health that epidemiologic transition theory is useful for conceptualizing: the increased incidence of chronic inflammatory diseases (CIDs), such as allergic and autoimmune diseases, and the emergence and reemergence of infectious disease.

**Results:**

Situating these trends within epidemiologic transition theory, we explain the rise in CIDs with the hygiene hypothesis and the rise in emerging and reemerging infections with the concept of a third epidemiologic transition.

**Conclusions:**

Contextualizing these trends within epidemiologic transition theory reveals implications for clinical practice, global health policies, and future research within epidemiology.

For the past several decades, the model of epidemiologic transitions has served as a guiding framework for understanding relationships between patterns of human health and disease and economic development (1). As originally proposed by Omran (2), an epidemiologic transition is a trend wherein a high burden of mortality from infectious disease—primarily epidemic ‘childhood’ diseases such as pertussis and measles—is replaced by one of chronic and non-communicable diseases (NCDs), such as cardiovascular disease, cancer, and diabetes. Omran's ‘classic’ model was originally formulated to capture the changes in cause-specific mortality that followed the Industrial Revolution in the United States and in Western Europe. However, in a modified form, the transition is ongoing in many developing low- and middle-income countries (LMICs), which carry a high burden of mortality from infectious diseases and NCDs (3).

Recently, scholars have placed the classic transition within an expanded evolutionary framework. This recognizes a ‘first’ transition coincident with the Neolithic period and the agricultural revolution and a ‘third’ transition of emerging and reemerging infectious diseases occurring in the modern era (1). In this expanded framework, Omran's classic transition becomes the ‘second epidemiologic transition’, as it is referred to here.

Epidemiologic transition theory provides a model for the dynamics among economic, social, demographic, and ecological factors and the evolution and spread of disease (4), explaining major trends in the human disease-scape and granting insight into ultimate causes of a given trend and therefore potential solutions (5). Consequently, it has become paradigmatic in public health policy (3, 6–9), demography (10, 11), biological and medical anthropology (12–14), and economics (15). However, it has had much less impact in epidemiology (5, 16, 17). This is because epidemiologists are generally concerned with the study of one or a few specific diseases within restricted time frames. Identifying a causal pathogen or characterizing a novel condition necessitates attention to its specific properties, such as statistical risk factors or disease ecology. This epidemiological approach translates into conceptualizing diseases, including emerging diseases, as singular entities attributable to more immediate and proximate causes, not as components of broader health trends attributable to longer-term and ultimate causes (5). However, as we discuss here, Omran's classic theory and its modifications can help to broaden the epidemiologist's understanding of the complex, multiple dimensions of health and disease over time, revealing potential proximate as well as ultimate causes, prevention strategies, and predictions of future epidemiologic trends and, in doing so, contribute to improving population health (5, 16).

Moving beyond Omran's original formulation, this paper critiques and modifies the theory of epidemiologic transitions, highlighting some of the ways that an understanding of epidemiologic transitions can benefit theory and practice in epidemiology. We also focus on two broad contemporary trends in human health that are relevant to epidemiologic transition theory: the increased incidence of chronic inflammatory diseases (CIDs), such as allergic and autoimmune diseases, and the emergence and reemergence of infectious diseases. We situate these trends within an expanded epidemiologic transition theory, employing the hygiene hypothesis to explain the rise in CIDs and the concept of a third epidemiologic transition to explain the rise in emerging and reemerging infections. We further discuss the implications that this approach generates for preventive medicine, global health policies, and future research in epidemiology.

## Present investigation

### The theory of epidemiologic transitions

**Fig. 1:**
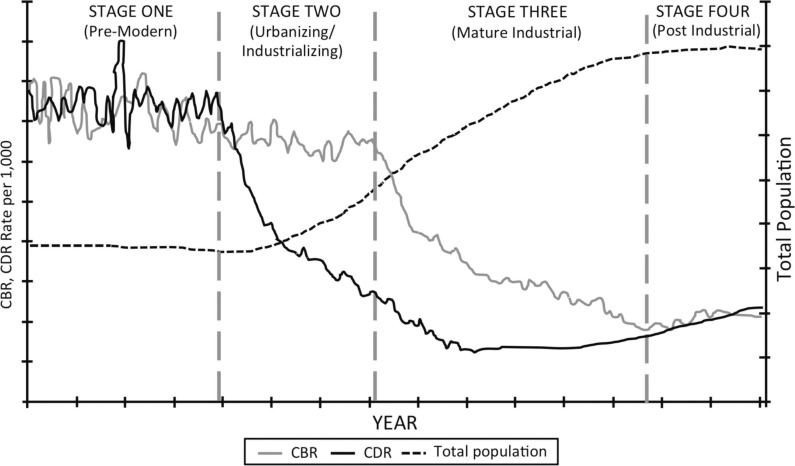
The demographic transition model (Source: K. Montgomery).

Epidemiologic transition theory models the changes in cause-specific mortality that accompanied the industrialization-associated demographic transition, the declines in mortality and fertility, and the resulting population growth (18) (see Fig. 1). Demographic transition theory is a simplified, descriptive, multistage model of this transition (14). Stage 1 is largely preindustrial and features high mortality and fertility, variable but generally low life expectancy, and slow but stable population growth interrupted by large periodic fluctuations in mortality (crisis mortality). Stage 2 involves declining ‘normal’ mortality but persistently high fertility. Stage 3 features continued declines in normal mortality as well as fertility, increased life expectancy, and more sustained population growth. Declining normal mortality began in the mid-nineteenth century in western and northern Europe and the United States and around 1920 in LMICs, such as Chile. Crisis mortality continued into the twentieth century, perhaps ending only with the 1918–1919 Spanish influenza outbreak (14). Stage 4 features low, still declining mortality, as observed in developed countries after World War II (19). Mortality declines continue, especially among the elderly, but slowly, likely due to already low mortality among infants, children, and young adults (14).

As originally formulated (2), the epidemiologic transition theory builds on demographic transition theory with a consideration of changes in cause specific mortality across four stages. Stage 1, the ‘Age of Pestilence and Famine’, is preindustrial and is characterized by high frequencies of epidemic infectious disease and crisis mortality. Stages 2 and 3, the ‘Age of Receding Pandemics’, involve a change from epidemic to endemic infectious disease and decreasing crisis mortality. Stage 4, the ‘Age of Degenerative and Man-Made [*sic*] Diseases’, ushers in a high burden of NCDs, the result of age-related degenerative processes and anthropogenic factors including environmental hazards and nutritional and behavioral patterns associated with industrialization and urban living. The neat dichotomy between infections and NCDs is blurred by the chronic course of some infections, such as tuberculosis, and increasing recognition of the role of infection and inflammatory processes in many chronic conditions, such as cervical cancer (20) and coronary heart disease (21). However, rather than precluding the use of transition theory, this complication highlights the need to consider the historical and evolutionary relationships between humans and pathogens to understand current patterns of human health.

### Modifications and critiques of epidemiologic transition theory

Multiple modifications have been made to Omran's original theory. Omran (2), attempting to accommodate variations from the theory, initially characterized three models for the progress of the transition. The ‘classical model’, featuring slow progression from high to low mortality and fertility, occurred in Western Europe, and was driven by economic development and advances in sanitation, public health, and medical knowledge. The ‘accelerated model’, seen in Japan, features more rapid progress, purportedly driven by the same factors. The ‘contemporary model’ applies to LMICs, wherein mortality has declined but fertility remains high and NCDs do not yet constitute the primary epidemiologic burden. Beyond the expansions suggested by Barrett and colleagues (1), Olshansky and colleagues have added a fourth stage, the ‘Age of Delayed Degenerative Diseases’, which recognizes a shift in NCDs to the elderly even as life expectancy increases, and a fifth, encompassing morbidity and mortality from HIV/AIDS and other emerging infectious diseases (22, 23). Graziano (24) has proposed an alternative fifth stage, the ‘Age of Obesity and Inactivity’, in which increasing levels of weight gain and obesity alter the pattern of NCDs among the elderly (represented by Olshansky and Ault's fourth stage).

From anthropological and epidemiological perspectives, the original epidemiologic transition theory has several shortcomings. The proposed explanations are largely speculative, lacking reliable data or relying on small sample sizes that are not likely to be generalizable (16). Many have also argued that the theory insufficiently addresses social factors, such as poverty (12); the differential progress of the transition within and across various demographic subgroups—such as in relation to sex, gender, race, and location (12, 25); and the profound contemporary impact of emerging and reemerging infectious diseases, such as HIV/AIDS and multidrug resistant tuberculosis (MDR TB)(12, 25, 26). It also fails to differentiate adequately between the *risk* of dying from any given cause or set of causes and the *proportion* of overall mortality due to various causes (14, 25). Additionally, by focusing on mortality, the theory largely neglects issues of morbidity, disability, and quality of life. This approach is sharply divergent from the increasing use of more holistic definitions of health and the use of broader measures of life expectancy, such as disability adjusted life years (DALYs), by health researchers and organizations (16). Saliently, Caldwell (6: 160) and others have also critiqued Omran's models for failing to recognize ‘the global nature and historical sequence of the mortality transition as it spread’, positing instead that each society exhibits its own particular model. Despite these issues, however, the theory remains profoundly useful for investigating variation in patterns over time and among locations, for conceptualizing historical patterns, and for predicting future trends (17).

### Epidemiologic transition theory and epidemiology

Although epidemiology largely maintains its focus on single disease conditions and proximate causes, the rise of the socioecological model in epidemiology represents an opportunity for integrating epidemiologic transition theory into epidemiologic theory and practice. This model replaces the earlier ‘epidemiologic triad’ and ‘causal web’ models of disease risk, which paid inadequate attention to the multidimensional quality of factors that affect health and disease (27), particularly the cultural, political, and economic factors that act as ultimate causes of disease (28, 29). Instead, the socioecological model recognizes that a broad array of systems and interrelated determinants of health exist, acting either synergistically or antagonistically (16). The model vertically expands the domain of epidemiological studies ‘upward’ to incorporate biological, behavioral, mental, social, and environmental systems and factors (such as policy and economic environments) as well as ‘downward’ to the molecular and genetic levels. Additionally, it extends the domain horizontally to consider trends over time, ranging from the developmental issues addressed by life course epidemiology to evolving associations among the various levels (16: 6).

Epidemiologic transition theory provides the broad, longitudinal, and historical perspectives that the early models lacked (16). In epidemiology, the increasing adoption of and reliance upon socioecological models has also opened up a spectrum of possibilities for investigations of disease risk that include social, cultural, policy, and economic factors, as well as how these factors have changed over time (5, 16). The unique capacity of epidemiologic transition theory to identify ultimate causes in disease risk means that transition theory models can be usefully incorporated into socioecological models in epidemiology. As Fleischer and McKeown (16: 10) discuss, such incorporation would be particularly useful for social epidemiologists because it would shed additional light on the ways in which ‘upstream’, global determinants differentially impact the health-states of vulnerable, disadvantaged populations. Although the focus on singular disease conditions, their risk factors, and proximate causes is useful in some investigations, epidemiologists are gradually recognizing that addressing patterns of health-states might yield greater insights into more upstream, shared determinants and therefore have a profound impact upon population health (16).

### Contemporary trends in health addressed through epidemiologic transition theory

#### CIDs and emerging and reemerging infectious disease

Epidemiological transition theory can be usefully applied to a variety of broad trends in patterns of health and disease in contemporary human populations. Here we focus on two specific trends: the increased incidence of CIDs and the emergence and reemergence of infectious diseases. We use these examples to demonstrate how the interpretive lens and attention to ultimate causes generated by epidemiologic transition theory can be used to inform clinical practice, global health policies, and future research within epidemiology.

#### The hygiene hypothesis and the rise of CIDs

Since the 1950s, numerous researchers have noted increased rates of CIDs, namely allergic and autoimmune diseases, in many developed nations (30). These include allergic diseases, such as asthma and atopic dermatitis, and autoimmune diseases, such as multiple sclerosis (MS) and inflammatory bowel disease. For instance, the prevalence of atopic dermatitis has doubled or tripled in developed countries over the past three decades, affecting 15 to 30% of children and 2 to 10% of adults (31). Part of these increases may be attributable to detection bias via improved diagnoses and access to medical resources in many locations, but this does not entirely explain the rapid rise in the incidence of these conditions, particularly those such as MS that are easily diagnosed (32).

Many epidemiologists have noticed that the increase in CIDs is concomitant with a decrease in epidemic infections that occurred during the second epidemiologic transition. However, rather than highly virulent pathogens, such as smallpox or measles (33), the phenomenon has been attributed to the diminished exposure to environmental microorganisms (specifically helminthic parasites, chronic viruses and nonlethal bacterial infections, environmental saprophytes) and to declines in the mass and diversity of gut microbiota, which also occurred as part of the second transition (34). Situated within epidemiologic transition theory, these observations have given rise to the ‘hygiene hypothesis’, which claims that in developed nations, diminished childhood exposure during childhood or even diminished prenatal (maternal) exposure (35) to these microorganisms has resulted in immunoregulatory failure, manifesting as an increased incidence of CIDs. According to this theory, prior to industrialization, humans lived in a state of ‘evolved dependence’ with these microbes—our ‘old friends’—that was critical for successful immunological functioning, specifically the development of an immunomodulatory response that maintains tolerance of self-antigens and abrogates autoimmune diseases (34). In effect, the lifestyle changes—sanitary improvements, pasteurization, use of antibiotics, and improved hygiene—that contributed to the second transition may have produced a substantial trade-off in health and quality of life, with developed nations exchanging a high burden of infectious disease for a higher burden of CIDs (36).

Using the hygiene hypothesis to identify potential ultimate causes of CIDs has several direct implications for epidemiology. Although the identified causes are broadly environmental and evolutionary, they directly translate into proximate causes and therefore specific risk factors and targets for preventive medicine. Informed by the hygiene hypothesis, researchers have identified causal relationships among lifestyle changes, infectious burden, and the incidence of allergic and autoimmune diseases. They continue to investigate these dynamics using animal models of autoimmune and allergic diseases and, to a lesser extent, clinical intervention studies (32). For example, the incidence of spontaneous type 1 diabetes (T1D) is directly correlated with the sanitary conditions of animal facilities for non-obese diabetic (NOD) mice, with a low infectious burden translating into a high T1D incidence (37). Another study found that intranasal exposure of pregnant mice to cowshed derived, nonpathogenic bacterium, *Acinetobacter lwoffii* F78, protected against the development of experimental asthma in their progeny (35). Other researchers have found that in humans, intentional infection with the swine-derived helminth, *Trichuris suis*, ameliorated symptoms in patients with active Crohn's disease as well as ulcerative colitis (38, 39). Although research findings are not yet determinative—for instance, helminth eradication has been found to increase atopic skin sensitization (40) while improving asthma symptoms in the same population (41)—studies such as these suggest that approaching the broad trend of increased incidence of CIDs through an evolutionary, historical lens generates findings that can be directly applied to improving population health. Research focused on determining which types of microbial exposures exert a protective effect on developing allergic sensitization (and when during the life course they occur) can translate into improved identification of risk factors, targeted strategies for disease prevention, and foci for preventive medicine.

A practical outcome of this approach can be found in the recent proposed ruling by the Food and Drug Administration (FDA) that manufacturers of antibacterial soap that contains the chemicals triclosan and triclocarban demonstrate that they are safer and more effective than soap and water. This ruling is the product of years of mounting fears that the chemicals in the soaps, the use of which has greatly proliferated in the past few decades, may disrupt normal development of the reproductive system and metabolism and promote drug-resistant infections, among other issues (42). This action reflects increasing public health awareness that interfering with long-standing balances between humans and nonpathogenic microbes in their environments can have pervasive and substantial health effects.

#### Addressing the ancient determinants of emerging infections

One of the chief advantages of an expanded framework of epidemiologic transitions, such as that described by Barrett and colleagues (1), is that it allows us to backtrace the ultimate determinants of current infections over long stretches of time back into ancient history and prehistory. Looking back to the Neolithic period, changes in subsistence, settlement, and social organization associated with the agricultural revolution created conditions that selected for the emergence of acute, epidemic, ‘crowd’ infectious diseases, such as smallpox, measles, and pertussis, which were among the chief causes of human morbidity and mortality up until the second epidemiologic transition (1). Agriculture allowed for the production of high calorie foods at the cost of dietary diversity (43), and agricultural communities that rely heavily on a few crops often experience compromised nutrition that can predispose them to infection (44, 45). In addition, domesticated animals served as the source for many novel human pathogens, such as measles and smallpox, as did the expansion of farmers into new terrain suited to the transmission of diseases such as malaria and yellow fever (46). The transition to large and densely settled societies also allowed for the ongoing transmission of more virulent and shorter-lived infections, such as crowd diseases, within and between human populations. Furthermore, the creation of social hierarchies led to the unequal distribution of basic resources for healthy living, thereby creating reservoirs for new and recurring infections in impoverished communities (12, 47).

Today, we see the same themes of subsistence, settlement, and social organization in the emergence and reemergence of infectious diseases. The hyperurbanization of domesticated animals, combined with the use of antibiotic growth factors, is contributing to increases in selective conditions for the entry of zoonotic pathogens from animal to human populations (48). Human settlements have consolidated to the point that the majority of the global population now lives in urban environments with ample opportunities for ongoing disease transmission (49). Additionally, globalization has linked the health problems of impoverished communities with other populations throughout the globe, in developed nations and LMICs, such that humans can now be said to live within a single infectious disease ecology. The scale and speed of human activities have greatly increased since the Neolithic, but the activities themselves are qualitatively the same, despite the novelty of some pathogens.

This longer term, evolutionary perspective has practical applications for the prevention and control of new and drug-resistant infections. Recognizing that the agricultural revolution brought new infections into human populations through increased exposure to animals, researchers have examined the entry of novel contemporary pathogens through human settlement in uninhabited wilderness areas and subsistence practices such as bushmeat hunting. Research has shown that bushmeat handling and consumption of nonhuman primates in particular has provided a highly effective pathway for the transmission of zoonotic pathogens into human populations, both in the past and today (50); several infections in humans, such as HIV/AIDS (via simian immunodeficiency viruses [SIVs]) (51) and a wide variety of human T-lymphotropic viruses (HTLVs), which are associated with leukemia, lymphoma, and HTLV-associated myelopathy (52), have been linked to pathogens present in nonhuman primates. Understanding these relationships and the pathological consequences of interspecies interactions has substantial implications for public health. As in Neolithic times, the wide array of subsistence practices that increase exposure between humans and animals increases the probability that nonhuman pathogens will, through a series of evolutionary steps, evolve the ability to infect humans, and later, evolve the capacity for sustained transmission within and between human populations. Recognizing these dynamics helps to inform international economic and environmental policies and technological innovations aimed at reducing these exposures (53).

Similarly, researchers can extend lessons from the intensification of agriculture to preventing, or at least reducing, the evolution of drug resistant infections. The FDA announced in December 2013 that it was phasing out the use of antibiotics as growth promoters in cows, pigs, and chickens (54). Although this policy is voluntary for drug manufacturers and subject to loopholes, it represents one of the first attempts by the U.S. government to address one of the ultimate causes driving the third epidemiologic transition, specifically the reemergence of previously controlled infectious diseases. The European Union has long since recognized this issue, having passed regulations against the use of human antibiotics, and their analogs, as growth factors in livestock (55). This policy change and the research underlying it reflect an increased understanding in the public health community that drug resistance not only poses a large and growing threat to human health but that this trend fits into a larger evolutionary pattern of selective relationships between humans, animals, and pathogens. Ten thousand years ago, domestication of animals gave human populations zoonotic infections; in the present, our interactions with domesticated animals are conferring drug resistant infections upon us.

### Conclusion: global health and the third epidemiologic transition

The current trends of novel, virulent, and drug resistant infectious diseases represent a third epidemiologic transition in human disease. To some extent, this can be seen as a convergence of disease patterns associated with the two previous epidemiologic transitions. As with the first transition, physical and social environments are once again selecting for the emergence and transmission of acute epidemic infections in human populations. As with the second transition, NCDs continue to rise in populations rich and poor, in developed nations and LMICs alike. In a combination of these two trends, acute and chronic diseases are leading to the emergence of global syndemics, wherein the interaction between two or more health conditions—such as among diabetes, cardiopulmonary disease, and severe acute respiratory syndrome (SARS)—has a multiplicative rather than additive impact on human well-being (56).

An expanded theory of epidemiologic transitions not only deepens understanding of these global health trends, it also informs policies and programs for prevention, detection of novel conditions and pathogens, identification of risk factors, and control of diseases. Shifting from primarily vertically organized programs aimed at single diseases and proximate causes, epidemiologists and other health researchers can develop complementary horizontally based programs aimed at combinations of infectious and chronic diseases and the upstream determinants that they have in common. The U.S. Centers for Disease Control and Prevention (CDC) has begun this process with the merging of previously independent programs and divisions within its organization (57). Similarly, observers of polio eradication programs, for example, have increasingly begun to recognize that treatment and vaccination cannot be successfully implemented without addressing broader health determinants, such as those responsible for the first and second epidemiologic transitions (58). Applying these lessons to current global health challenges may not eradicate all human diseases, but it could generate and marshal support for novel, broadscale interventions that target the ultimate causes for many important health conditions. At that point, we hope to write optimistically about the positive lessons evident a fourth epidemiologic transition.

**Main findings**

- Integrating epidemiological transition theory into theory and practice in epidemiology can broaden epidemiologists’ understanding of the complex, multiple dimensions of health and disease over time. This can reveal potential proximate and ultimate causes, prevention strategies, and predictions of future epidemiologic trends, therefore contributing to improvements in population health.
- Contextualizing disease trends, such as the increased incidence of chronic inflammatory diseases (CIDs), including allergy and autoimmune diseases, and the emergence and re-emergence of infectious disease, within epidemiological transition theory, and attending to historical and evolutionary relationships between humans and pathogens, has direct implications for clinical practice, global health policies, and future research within epidemiology.

**Key messages for action**

- Situating the increased incidence of CIDs within the hygiene hypothesis, a theory closely associated with epidemiologic transition theory that ties the trend to immunoregulatory failure brought on by decreased early life exposure to non-pathogenic environmental microbes, can be used to identify specific risk factors, targeted strategies for disease prevention, and foci for preventive medicine in global efforts to reduce CIDs.
- Addressing the emergence and re-emergence of infectious disease, such as HIV/AIDs and MDR-TB, within long term evolutionary perspectives can reveal the ancient and prehistoric ultimate determinants of current infections, generating practical applications for the prevention and control of new and drug-resistant infections, such as attendance to the role of bush-meat and environmental degradation in the emergence and spread of disease.
- The critical insight from epidemiologic transition theory that interference with long-standing balances between humans and non-pathogenic microbes in their environments can have pervasive and substantial health effects has directly informed recent action in public health, such as the FDA's proposed rulings discouraging the use of antibiotics in livestock feed and antibacterial compounds in soaps.
